# Prevalence and factors associated with sleep deprivation in nursing technicians working in shifts

**DOI:** 10.1590/0034-7167-2025-0143

**Published:** 2026-11-06

**Authors:** Renato Canevari Dutra da Silva, Ana Carolina Moraes de Oliveira, Nicole Jorge Teixeira, Elton Brás Camargo, Heloísa Silva Guerra, Lara Cândida de Sousa Machado, Yasmin Koba Canevari Dutra, Antônio Augusto Schäfer

**Affiliations:** I Universidade de Rio Verde. Rio Verde, Goiás, Brazil; II Centro Universitário Padre Albino. Catanduva, São Paulo, Brazil; III Universidade do Extremo Sul Catarinense. Criciúma, Santa Catarina, Brazil

**Keywords:** Nurse Practitioners, Sleep Deprivation, Sleep Disorders of Circadian Rhythm, Occupational Health, Shift Work Schedule., Enfermeras Practicantes, Privación de Sueño, Trastornos del Sueño del Ritmo Circadiano, Salud Laboral, Horario de Trabajo por Turnos.

## Abstract

**Objectives:**

to estimate the prevalence of sleep deprivation among nursing technicians working in hospital shifts and identify the associated factors.

**Methods:**

a cross-sectional study was conducted at a municipal university hospital in Goiás, Brazil, between March and May 2022, with 198 participants. Sleep deprivation was assessed using the Pittsburgh Sleep Quality Index, defined as less than five hours of sleep per night. Multivariate analysis was used to identify factors associated with sleep deprivation.

**Results:**

the prevalence of sleep deprivation was 43.9%. The main factors associated with sleep deprivation included prolonged daily work hours (OR=3.60/p=0.001), inadequate consumption of fruits, vegetables and greens (OR=2.25/p=0.024), coffee consumption (OR=3.14/p=0.031), high Body Mass Index (OR=1.27/p=0.001) and presence of anxiety symptoms (OR=6.58/p=0.001).

**Conclusions:**

intervention strategies should consider occupational and mental health factors to improve nursing technicians’ sleep quality.

## RESUMEN

**Objetivos:**

estimar la prevalencia de la privación del sueño entre los técnicos de enfermería que trabajan en turnos hospitalarios e identificar los factores asociados a esta condición.

**Métodos:**

este estudio transversal se realizó en un hospital universitario municipal de Goiás, Brasil, entre marzo y mayo de 2022, con 198 participantes. La privación del sueño se evaluó mediante el Pittsburgh Sleep Quality Index, definido como menos de cinco horas de sueño por noche. Se utilizó un análisis multivariado para identificar los factores asociados con la privación del sueño.

**Resultados:**

la prevalencia de la privación del sueño fue del 43,9%. Los principales factores asociados a la privación del sueño incluyeron jornadas laborales prolongadas (OR=3,60/p=0,001), consumo insuficiente de frutas, verduras y hortalizas (OR=2,25/p=0,024), consumo de café (OR=3,14/p=0,031), Índice de Masa Corporal elevado (OR=1,27/p=0,001) y presencia de síntomas de ansiedad (OR=6,58/p=0,001).

**Conclusiones:**

las estrategias de intervención deben tener en cuenta los factores laborales y de salud mental para mejorar la calidad del sueño de los técnicos de enfermería.

## INTRODUCTION

Sleep is a natural state of the body and mind that, in addition to conserving energy, plays essential roles in the organism, such as stimulating the synthesis of proteins and hormones, reducing oxidative stress, removing metabolic waste from the brain, and strengthening the immune system^(1)^. During sleep, the body’s systems enter an anabolic state, which in turn promotes the restoration of the nervous, immune, skeletal, and muscular systems, essential for maintaining memory, cognition, mood, and endocrine functions^(2)^.

The growth and diversification of work in the global economy have increased the need to maintain high levels of productivity, often at the expense of rest^(3)^. In this scenario, shift work emerged as a solution to ensure the continuity of operations, especially in sectors that require uninterrupted operation. This model is characterized by the organization of work in which workers successively occupy the same positions at different times over a given period, such as days or weeks^(4)^. While efficient in meeting production demands, this model poses challenges to workers’ health and well-being, including sleep cycle disturbances and compromised physical and mental health^(3,4)^.

Among healthcare professionals, especially nurses, sleep deprivation is a frequent condition due to long working hours and shift work^(5)^. During hospitalization, the nursing staff remains on duty 24 hours a day, providing intensive care to both patients and their families. This exhausting workload makes these professionals particularly vulnerable to changes in their quality of life, especially those working night shifts, as the psychophysiological strain is more pronounced because they perform their duties while the body is naturally at rest^(6)^.

Although previous research has shown that professionals such as nurses who work in shifts face a significant decline in sleep quality, a higher prevalence of metabolic and cardiovascular disorders, stress, and occupational errors^(7,8)^, there are still few investigations that extend this analysis to other work groups exposed to similar shifts.

Widely recognized epidemiological studies indicate that the ideal sleep duration for healthy adults should range between seven and nine hours per night, with an adequate range considered to be between six and ten hours, depending on individual needs, age, lifestyle, and health conditions^(9,10)^. Chronic sleep deprivation, or the constant disruption of the sleep cycle, can have significant implications for physical and mental health, contributing to an increased risk of cardiovascular and metabolic diseases, cognitive disorders, and a decreased quality of life^(11)^.

However, when observing healthcare professionals’ reality, especially nurses’ reality, this recommendation is not commonly met. A previous study showed that the average number of hours nurses sleep is only six hours per night, an amount lower than the minimum recommended for well-being and adequate performance^(12)^. This occurs due to the nature of long and irregular work schedules, especially those involving night shifts, which make it difficult to maintain adequate sleep patterns and, consequently, negatively impact health and professional performance^(4,5)^.

## OBJECTIVES

To estimate the prevalence of sleep deprivation among nursing technicians working hospital shifts and to identify factors associated with this condition.

## METHODS

### Ethical aspects

This study rigorously followed the ethical principles established for research with human subjects. All participants were invited to participate voluntarily and were duly informed about the objectives, procedures, potential risks, and benefits of the study. Before data collection, each professional signed the Informed Consent Form, which guaranteed their agreement and understanding of the research, as stipulated by Resolution 466/12 of the Brazilian National Health Council.

Furthermore, the study was submitted to and approved by the Universidade de Rio Verde Research Ethics Committee, under the Certificate of Presentation for Ethical Consideration, in compliance with current ethical and scientific regulations. The confidentiality and privacy of participants’ information were preserved at all stages of the research.

### Study design, period, and setting

This cross-sectional and analytical study was conducted in accordance with STrengthening the Reporting of Observational studies in Epidemiology methodology guidelines. It investigated the prevalence of sleep deprivation among nursing technicians working hospital shifts and its relationship with socioeconomic, professional, behavioral, nutritional conditions, and sleep-related factors. Data collection was carried out using structured individual questionnaires, with the aim of obtaining detailed information about participants.

The research was conducted at a municipal university hospital in a city in Goiás state, in a referral hospital unit, between March and May 2022. The hospital offers medium and high complexity care and has a qualified multidisciplinary team. The unit’s structure includes beds for clinical and surgical hospitalization, as well as emergency services, making it a strategic setting for the study of professionals subject to different work shifts.

### Population or sample; inclusion and exclusion criteria

The study population consisted of nursing technicians working in a hospital setting, totaling 218 professionals. The sample was non-probabilistic, based on convenience, and included all nursing technicians present at the hospital during the data collection period. Professionals of both sexes, aged between 18 and 60 years, were included, while those with less than six months of uninterrupted activity were excluded. In total, 198 volunteers participated, distributed among the three work shifts.

### Study protocol

Participants were approached during their rest periods in a room provided by the hospital administration, offering privacy and comfort for the administration of the research questionnaire.

The questionnaire used was standardized, pre-tested, and self-administered, covering demographic, socioeconomic, nutritional, behavioral, professional, and mental health and sleep-related variables.

The outcome variable was sleep deprivation, measured using the sleep duration component of the Pittsburgh Sleep Quality Index (PSQI). This component was determined through questions investigating usual bedtime, wake-up time, and time required to fall asleep, allowing for the calculation of the mean number of hours slept per night^(13)^. Sleep deprivation was categorized according to criteria widely adopted in the literature: individuals with an average sleep time of less than five hours per night (< five hours) were classified as sleep-deprived, while those with a sleep time equal to or greater than five hours (≥ five hours) were considered to have adequate sleep^(14)^.

Exploratory variables were grouped into thematic categories for organizational and analytical purposes: (i) demographic, socioeconomic, and professional; (ii) nutritional; (iii) behavioral; (iv) mental health; and (v) sleep-related. (i) Demographic, socioeconomic, and professional: sex (male; female), marital status (with partner; without partner), education level (< 15 years of study; ≥ 15 years), mean years in the profession, and daily working hours (≤ 12 hours; > 12 hours). (ii) Nutritional: consumption of fruits, vegetables, and greens (> five portions/day - adequate; < five portions/day - inadequate)^(15)^, consumption of fast food (yes; no), self-reported weight and height and Body Mass Index (BMI). (iii) Behavioral: alcohol consumption (yes: has consumed; no: has never consumed or has stopped drinking), coffee consumption (yes; no), smoking (current smokers; non-smokers/former smokers), level of internet dependence (mild; moderate/high), assessed by the Internet Addiction Test^(16)^, and risk of alcohol dependence (low risk; risk of dependence), categorized by the Alcohol Use Disorders Identification Test scale. (iv) Mental health: symptoms of depression, anxiety and stress were assessed using the Depression, Anxiety and Stress Scale-21^(17)^ and categorized as low or high risk, in addition to self-perception of health (good/very good; poor/very poor). (v) Sleep-related: sleep quality assessed by the PSQI (good; poor)^(13)^ and excessive daytime sleepiness (EDS), measured by the Epworth Sleepiness Scale and categorized as presence (> 10) or absence (< 10)^(15,18)^.

### Analysis of results and statistics

Data entry was performed using EpiData 3.1 software, with double entry to minimize errors. Data validation and analysis were conducted using Stata 15.0 software. Initially, the information was organized in Microsoft Excel^®^ 2013, and statistical processing was performed using the Statistical Package for the Social Sciences^®^.

Descriptive statistical analyses were performed to characterize the sample and the study variables. Absolute and relative frequencies were used. To identify the factors associated with sleep deprivation, bivariate and multivariate logistic regressions were applied. Initially, each independent variable was analyzed in isolation in the bivariate logistic regression to verify its relationship with sleep deprivation. Variables with a p-value less than 0.2 were selected to compose the multivariate model.

Subsequently, multivariate logistic regression was conducted using the forward method. The independent variables selected in the previous stage were progressively included (p < 0.2). This approach made it possible to assess the combined effect of variables on sleep deprivation, considering potential confounding factors. Association estimates were expressed as Odds Ratios (OR) with their respective 95% Confidence Intervals (95%CI). To determine the statistical significance of the associations in the multivariate model, the criterion of p < 0.05 was adopted.

## RESULTS

The sample consisted of 198 participants, mostly women (95.5%), with a mean age of 37.2 years. Table 1 presents data on sleep deprivation and its association with sociodemographic, professional, nutritional, behavioral, mental health, and sleep-related characteristics of nursing technicians. The majority of individuals reported being in a stable relationship (60.6%). Moreover, 68.2% had up to 15 years of education. The mean length of time in the profession was 7.5 years. Regarding eating habits, 59.1% of participants reported adequate consumption of fruits, vegetables, and greens, while 68.2% consumed fast food. The prevalence of alcohol consumption was high, with 69.7% of participants reporting the habit, and 83.3% consumed coffee regularly. The majority of participants (93.9%) were smokers or former smokers, and 62.1% were overweight or obese (BMI ≥ 25). In addition, 42.4% of participants reported symptoms of anxiety, and 12.1% presented depressive symptoms. Sleep quality was predominantly poor, with 93.9% of participants reporting this condition. The prevalence of sleep deprivation among participants was 43.9%, highlighting the magnitude of this problem in the daily lives of these professionals.

**Table 1:** Frequency of sleep deprivation and its association with sociodemographic, professional, nutritional, behavioral, mental health, and sleep-related characteristics of nursing technicians, Goiás, Brazil, 2022

Variables	Total	Sleep deprivation	
	n (%)	OR (95%CI)	*p* value
Sex			
Female	189 (95.5%)	1	0.174
Male	9 (4.5%)	2.67 (0.65-10.98)	
Marital status			
With partner	78 (39.4%)	1	0.211
Without partner	120 (60.6%)	1.45 (0.81-2.59)	
Education level			
Under 15 years	135 (68.2%)	1	0.476
15 years and above	63 (31.8%)	1.24 (0.68-2.27)	
Years in employment	7.5±7.0	0.98 (0.95-1.03)	0.500
Hours/day worked			
≤ 12 hours	132 (66.7%)	1	0.035^*^
> 12 hours	66 (33.3%)	1.90 (1.05-3.47)	
Fruit, vegetable, and green consumption			
Adequate (> five servings/day)	117 (59.1%)	1	0.063
Inadequate (< five servings/day)	81 (40.9%)	1.72 (0.97-3.06)	
*Fast food consumption*			
Does not consume	63 (31.8%)	1	0.834
Consumes	135 (68.2%)	1.07 (0.58-1.95)	
Alcohol consumption			
No	60 (30.3%)	1	0.462
Yes	138 (69.7%)	1.26 (0.68-2.33)	
Coffee consumption			
No	33 (16.7%)	1	0.038^*^
Yes	165 (83.3%)	2.39 (1.05-5.45)	
Smoker			
No/former smoker	12 (6.1%)	1	0.524
Yes	186 (93.9%)	1.15 (0.75-1.76)	
BMI			
< 24.9	75 (37.9%)	1	0.008^*^
25 to 29.9 /> 30	123 (62.1%)	1.45 (0.25-1.81)	
Internet addiction level			
Mild	183 (92.4%)	1	0.200
Moderate/high	15 (7.6%)	2.02 (0.69-5.91)	
Risk of alcohol dependence			
Low risk	165 (83.3%)	1	0.038^*^
Risk of dependence	33 (16.7%)	0.42 (0.18-0.95)	
Depression			
No	174 (87.9%)	1	0.194
Yes	24 (12.1%)	1.72 (0.76-3.89)	
Anxiety			
No	114 (57.5%)	1	0.001^*^
Yes	84 (42.4%)	2.62 (1.47-4.66)	
Self-perceived health			
Good/very good	141 (71.2%)	1	0.741
Poor/very poor	57 (28.8%)	0.90 (0.48-1.68)	
Sleep quality (PSQI)			
Good quality	12 (6.1%)	1	0.001^*^
Poor quality	186 (93.9%)	3.29 (0.25-6.53)	
EDS			
Without EDS	117 (59.7%)	1	0.905
With EDS	81 (40.9%)	1.03 (0.58-1.83)	

*BMI - Body Mass Index; EDS - excessive daytime sleepiness; PSQI - Pittsburgh Sleep Quality Index; OR - Odds Ratio; 95%CI - 95% Confidence Interval;*

* Statistically significant association.

Multivariate analysis identified a number of factors associated with sleep deprivation, which, in turn, revealed significant relationships with several sociodemographic, behavioral, and health variables. Among the main determinants identified, the following stand out: daily work schedule, dietary pattern related to the consumption of fruits, vegetables and greens, coffee consumption, BMI, and the presence of anxiety symptoms. Although sex and the risk of alcohol dependence were also analyzed, these factors did not show a statistically significant association with sleep deprivation. Therefore, it is suggested that the influence of these aspects may be secondary in relation to other determinants (Table 2).

**Table 2:** Association of variables with sleep deprivation among nursing technicians in multivariate logistic regression analysis, Goiás, Brazil, 2022

Variables	Sleep deprivation	
	OR (95%CI)	*p* value
Sex		
Female	1	0.137
Male	3.41(0.68-17.17)	
Hours/day worked		
≤ 12 hours	1	0.001^*^
> 12 hours	3.60(1.72-7.56)	
Fruit, vegetable, and green consumption		
Adequate (> five servings/day)	1	0.024^*^
Inadequate (< five servings/day)	2.25(1.11-4.54)	
Coffee consumption		
No	1	0.031^*^
Yes	3.14(1.11-8.89)	
BMI		
< 24.9	1	0.001^*^
25 to 29.9/30	1.27(0.13-0.56)	
Risk of alcohol dependence		
Low risk	1	0.059
Risk of dependence	0.41(0.16-1.03)	
Anxiety		
No	1	0.001^*^
Yes	6.58(3.07-14.11)	
Sleep quality (PSQI)		
Good quality	1	0.001^*^
Poor quality	3.36 (1.23-5.57)	

*BMI - Body Mass Index; PSQI - Pittsburgh Sleep Quality Index; OR - Odds Ratio; 95%CI - 95% Confidence Interval;*

* Statistically significant association.

The daily work schedule was one of the factors most strongly associated with sleep deprivation. Individuals who worked more than 12 hours a day had a 3.6 times greater risk of suffering from sleep deprivation compared to those who worked up to 12 hours (OR = 3.60; 95%CI: 1.72-7.56; p = 0.001). This data suggests that prolonged work schedules have a significant impact on sleep quality.

Inadequate consumption of fruits, vegetables, and greens was also linked to sleep deprivation. Participants with an inadequate diet in this respect had a 2.25 times greater chance of experiencing sleep deprivation compared to those who consumed an adequate serving of fruits, vegetables, and greens (OR = 2.25; 95%CI: 1.11-4.54; p = 0.024), indicating that diet plays an important role in sleep well-being.

Coffee consumption was another significant factor, with those who consumed coffee having a 3.14 times greater risk of suffering from sleep deprivation compared to those who did not consume the beverage (OR = 3.14; 95%CI: 1.11-8.89; p = 0.031). This suggests that coffee consumption may negatively affect sleep, possibly due to the stimulating effect of caffeine.

Regarding BMI, participants with higher values showed a greater risk of sleep deprivation. Individuals with a BMI greater than 24.9 had a significantly higher chance of having sleep problems, with an OR of 1.27 (95%CI: 0.13-0.56; p = 0.001) compared to those with a BMI below 24.9.

The presence of anxiety was one of the factors most strongly associated with sleep deprivation, with anxious individuals showing a significantly higher OR (OR = 6.58; 95%CI: 3.07-14.11; p = 0.001). Furthermore, individuals with poor sleep quality showed a higher prevalence of sleep deprivation (OR = 3.36; 95%CI: 1.23-5.57; p = 0.001).

## DISCUSSION

The high prevalence of sleep deprivation observed among nursing technicians working shifts at this municipal university hospital in Goiás (43.9%) is consistent with previous studies documenting this condition among healthcare professionals, especially in critical care settings. Authors have reported prevalence rates of sleep deprivation ranging from 20% to 51%, similar to the 43.9% found in this study^(19,20)^. These findings reinforce the idea that occupational factors, such as night shifts, long working hours, and high emotional and cognitive demands faced by healthcare professionals, are significant determinants of sleep deprivation. The literature suggests that, due to work overload and emotional demands, healthcare professionals frequently experience sleep fragmentation and reduced rest quality^(21,22)^.

Sleep deprivation among healthcare professionals has been widely associated with several adverse outcomes, such as fatigue, reduced cognitive function, and an increased risk of medical errors^(23)^. A study identified that nursing professionals are among the most affected by sleep deprivation compared to other occupational categories, due to the nature of shift work and the constant need to adapt to irregular schedules. These findings highlight the importance of considering work structure, including shift flexibility and stress management, to reduce the prevalence of sleep deprivation and its effects on worker health and patient safety^(24)^.

The study revealed that working more than 12 hours a day was significantly associated with sleep deprivation. This finding is consistent with other research indicating that extended shifts and the need for night work negatively impact sleep patterns^(9,10)^. In particular, it is emphasized that chronic stress associated with hospital services, combined with long working hours, contributes to sleep fragmentation and increased sleep deprivation, leading to a vicious cycle of fatigue and exhaustion^(6)^.

Another relevant factor identified in this study was the association between inadequate consumption of fruits, vegetables, and greens and sleep deprivation, although with a trend towards statistical significance (p = 0.063). Diets rich in micronutrients such as magnesium, tryptophan, and B vitamins play a key role in regulating the circadian rhythm and producing sleep-related neurotransmitters like serotonin and melatonin^(25)^. Diets lacking in these nutrients can impair sleep quality. Therefore, nutritional interventions aimed at increasing the consumption of fruits, vegetables, and greens may be effective in improving sleep in this population^(26)^.

Caffeine consumption has also been identified as a factor significantly associated with sleep deprivation. Caffeine is known for its stimulating effect, which can prolong wakefulness and reduce sleep efficiency, especially when consumed at inappropriate times^(27)^. These findings are consistent with another study that documents the relationship between caffeine consumption and sleep disorders^(28)^. These findings reinforce the need for guidance on the impact of stimulant consumption on the well-being of healthcare professionals.

The finding that individuals with a BMI above 24.9 kg/m^2^ had a higher risk of sleep deprivation is consistent with the literature, which suggests a bidirectional relationship between excess weight and sleep disorders. Obesity can negatively affect sleep quality through physiological mechanisms, such as low-grade systemic inflammation and hormonal changes, which impact the regulation of the circadian rhythm and sleep homeostasis^(29)^. Furthermore, overweight and obese individuals often have a higher risk of sleep-disordered breathing, such as obstructive sleep apnea syndrome, which leads to sleep fragmentation and EDS^(30)^.

On the other hand, evidence suggests that sleep deprivation can contribute to weight gain, as it is associated with changes in energy metabolism and appetite, culminating in increased calorie intake, reduced satiety, and an imbalance in the secretion of regulatory hormones such as leptin and ghrelin^(31)^. Thus, the effect observed in this study may be part of a feedback loop, in which increased BMI contributes to poorer sleep quality, while sleep deprivation promotes weight gain.

The relationship between sleep deprivation and mental health was one of the most evident findings, with a significant association between the presence of anxiety and sleep deprivation. The literature indicates that the relationship between emotional disorders and sleep is bidirectional: anxiety can make it difficult to maintain sleep, and, in turn, sleep deprivation can exacerbate anxiety symptoms^(11)^. Nursing technicians working in medium and high-complexity hospitals are exposed to high emotional demands and constant stress, which may explain the high prevalence of emotional disorders in this population. Therefore, caring for the mental health of these professionals is crucial to improving sleep quality and, consequently, the quality of care provided.

The risk of alcohol dependence was inversely associated with sleep deprivation, with a lower prevalence of sleep deprivation among those at higher risk of dependence (p = 0.038). Although alcohol has a sedative effect, there is evidence that it impairs sleep quality by reducing REM sleep and increasing nighttime sleep fragmentation, which, in the long term, can worsen sleep disorders^(32)^.

The association between poor sleep quality and sleep deprivation observed in this study (OR = 3.36; 95%CI: 1.23-5.57; p = 0.001) reinforces previous findings indicating that individuals with poorer sleep quality are more likely to develop sleep deprivation. Literature suggests that poor sleep quality is often related to factors such as sleep fragmentation, difficulty initiating or maintaining sleep, and frequent awakenings, resulting in lower sleep efficiency and, consequently, insufficient total rest time for adequate physiological and mental recovery^(33)^.

Previous studies suggest that poor sleep quality may be associated with multiple factors, including stress, unfavorable environmental conditions, inadequate sleep habits, and excessive occupational demands^(34,35)^. Individuals with irregular sleep patterns, such as night shift workers or those subjected to long working hours, frequently report poorer sleep quality and greater sleep deprivation, which can contribute to metabolic changes, cognitive deficits, and worsened mental health^(23)^.

The relationship between sleep quality and sleep deprivation is bidirectional. Poor sleep quality can contribute to sleep deprivation by reducing rest efficiency and compromising physiological and cognitive restoration^(36)^. In turn, chronic sleep deprivation can exacerbate sleep fragmentation and reduce sleep depth, increasing the risk of disorders such as insomnia and EDS^(37)^.

In summary, the findings of this study highlight the importance of addressing sleep deprivation among nursing technicians from a multidisciplinary perspective, considering occupational, dietary, mental health, and behavioral factors. Implementing occupational health policies aimed at improving sleep quality, such as restructuring work schedules, promoting a balanced diet, and providing psychological support, can significantly contribute to reducing sleep deprivation and its impacts on health and work performance.

### Study limitations

This study has some limitations that should be considered when interpreting the results. Firstly, its cross-sectional nature prevents the determination of causal relationships between sleep deprivation and associated factors, which limits the findings to statistical associations. Furthermore, data collection was carried out through self-reported questionnaires, which are subject to recall and response bias, especially regarding subjective variables such as sleep quality and anxiety symptoms. Another limitation concerns the sample size, as the study was conducted in a single municipality in southwestern Goiás, which may restrict the generalizability of the findings to other populations of nursing technicians with different working conditions and socioeconomic contexts.

To overcome the limitations of this study, future research could adopt a longitudinal design, allowing for the establishment of causal relationships between the variables and sleep deprivation.

Despite these limitations, the study presents significant strengths, as the use of validated instruments to assess sleep and mental health strengthens data robustness, allowing for comparisons with previous studies. Furthermore, by addressing a professional group highly susceptible to sleep deprivation due to the nature of their work, the study contributes to understanding the factors that impact the health of these workers and provides support for the development of intervention strategies. Another positive aspect is the detailed analysis of multiple factors associated with sleep deprivation, which offers a comprehensive view of the occupational, behavioral, and emotional influences on this outcome.

### Contributions to nursing, health, or public policy

The findings of this study contribute to nursing practice by highlighting the high prevalence of sleep deprivation among nursing technicians working in hospital shifts and by emphasizing the occupational, nutritional, behavioral, and emotional factors associated with this condition. By identifying variables directly related to working hours, dietary patterns, consumption of stimulants, and mental health, the study provides practical support for planning strategies to promote worker health.

In clinical practice, the findings reinforce the need for educational and preventive programs focused on managing sleep deprivation, encompassing everything from organizational interventions regarding work hours to initiatives promoting balanced diets, moderation in stimulant consumption, and psychosocial support. Such measures can contribute not only to improving sleep quality, but also to the safety of the care provided and to reducing the physical and emotional strain on professionals.

At the institutional and public policy level, the data can guide managers in formulating guidelines that recognize sleep deprivation as a significant occupational health problem. Incorporating this topic into public health agendas promotes the creation of more sustainable work settings, with positive repercussions for professionals’ quality of life and, indirectly, for quality of healthcare. Furthermore, the results open up new avenues for research that deepen the understanding of the interaction mechanisms between occupational, nutritional, and psychological factors in sleep deprivation among healthcare professionals.

## CONCLUSIONS

This study revealed a high prevalence of sleep deprivation (43.9%) among participants, which is consistent with existing literature that frequently points to prolonged work hours, inadequate eating habits, mental health issues, and sleep quality as significant determinants of sleep deprivation.

The analysis demonstrated that daily work hours, consumption of fruits, vegetables, and greens, coffee consumption, BMI, and the presence of anxiety symptoms are factors significantly related to sleep deprivation. Among these, prolonged work hours were the most strongly associated factor, with individuals working more than 12 hours a day showing a significantly higher risk of suffering from sleep deprivation. Furthermore, an inadequate diet and coffee consumption have also been shown to be risk factors, reinforcing the importance of healthy eating habits and moderation in the consumption of stimulants.

The presence of anxiety was another prominent factor, with anxious individuals showing a substantially higher probability of suffering from sleep deprivation. This finding highlights the importance of psychological approaches, such as stress management and emotional well-being promotion, in managing sleep deprivation. Although sex and the risk of alcohol dependence did not show a significant association, the results suggest that these factors may have a secondary impact, being outweighed by other determinants more directly related to sleep health.

These findings indicate that the prevention and treatment of sleep deprivation should involve multifaceted strategies that consider not only working conditions, but also dietary habits, substance use, and psychological factors. Interventions that address these variables can contribute to improving nursing professionals’ sleep quality, health, and well-being, especially those working shifts in critical settings.

## Data Availability

The research data are available only upon request.
