# Supplementary material for: Prevalence and factors associated with sleep deprivation in nursing technicians working in shifts
Source: Rev Bras Enferm. 2026 Nov 6;79:e20250143. doi: 10.1590/0034-7167-2025-0143 (PMC13742972; doi:10.1590/0034-7167-2025-0143)
Supplement: Translation

# Translation: Prevalência e fatores associados à privação de sono em técnicos de enfermagem atuantes em turnos

**DOI:** 10.1590/0034-7167-2025-0143pt

**Authors:** Renato Canevari Dutra da Silva, Ana Carolina Moraes de Oliveira, Nicole Jorge Teixeira, Elton Brás Camargo, Heloísa Silva Guerra, Lara Cândida de Sousa Machado, Yasmin Koba Canevari Dutra, Antônio Augusto Schäfer

**Affiliations:** I Universidade de Rio Verde. Rio Verde, Goiás, Brasil; II Centro Universitário Padre Albino. Catanduva, São Paulo, Brasil; III Universidade do Extremo Sul Catarinense. Criciúma, Santa Catarina, Brasil

**Keywords:** Profissionais de Enfermagem, Privação do Sono, Transtornos do Sono do Ritmo Circadiano, Saúde Ocupacional, Jornada de Trabalho em Turnos.

## Abstract

**Objetivos:**

estimar a prevalência de privação de sono entre técnicos de enfermagem atuantes em turnos hospitalares e identificar os fatores associados a essa condição.

**Métodos:**

estudo transversal realizado em hospital universitário municipal de Goiás, entre março e maio de 2022, com 198 participantes. A privação de sono foi avaliada pelo *Pittsburgh Sleep Quality Index*, definida como menos de cinco horas de sono por noite. A análise multivariada foi utilizada para identificar os fatores associados à privação de sono.

**Resultados:**

a prevalência de privação de sono foi de 43,9%. Os principais fatores associados à privação de sono incluíram jornada de trabalho diária prolongada (OR=3,60/p=0,001), consumo inadequado de frutas, legumes e verduras (OR=2,25/p=0,024), consumo de café (OR=3,14/p=0,031), alto Índice de Massa Corporal (OR=1,27/p=0,001) e presença de sintomas ansiosos (OR=6,58/p=0,001).

**Conclusões:**

estratégias de intervenção devem considerar fatores ocupacionais e de saúde mental para melhorar a qualidade do sono dos técnicos de enfermagem.

## INTRODUÇÃO

O sono é um estado natural do corpo e da mente que, além de conservar energia, desempenha funções essenciais para o organismo, como estimular a síntese de proteínas e hormônios, reduzir o estresse oxidativo, remover resíduos metabólicos do cérebro e fortalecer o sistema imunológico^(1)^. Durante o sono, os sistemas do corpo entram em um estado anabólico, o que, por sua vez, promove a restauração dos sistemas nervoso, imunológico, esquelético e muscular, essenciais para a manutenção da memória, da cognição, do humor e das funções endócrinas^(2)^.

O crescimento e a diversificação do trabalho na economia global aumentaram a necessidade de manter altos níveis de produtividade, frequentemente à custa do descanso^(3)^. Nesse cenário, o trabalho em turnos surgiu como uma solução para garantir a continuidade das operações, especialmente em setores que exigem funcionamento ininterrupto. Esse modelo é caracterizado pela organização do trabalho em que os trabalhadores ocupam sucessivamente os mesmos postos em diferentes horários ao longo de um determinado período, como dias ou semanas^(4)^. Embora eficiente para atender às demandas produtivas, esse modelo impõe desafios à saúde e ao bem-estar dos trabalhadores, incluindo distúrbios no ciclo do sono e comprometimento da saúde física e mental^(3,4)^.

Entre os profissionais de saúde, especialmente os de enfermagem, a privação de sono é uma condição frequente devido às longas jornadas e à dinâmica de trabalho em turnos^(5)^. Durante a hospitalização, a equipe de enfermagem permanece em serviço por 24 horas, proporcionando cuidados intensos tanto aos pacientes quanto aos seus familiares. Essa carga de trabalho exaustiva torna esses profissionais particularmente vulneráveis à alteração da qualidade de vida, especialmente os que atuam durante a noite, pois o desgaste psicofisiológico é mais pronunciado, pois realizam suas atividades enquanto o organismo está naturalmente em repouso^(6)^.

Apesar de pesquisas prévias demonstrarem que profissionais como enfermeiros submetidos ao trabalho em turnos enfrentam significativa piora na qualidade do sono, maior prevalência de distúrbios metabólicos, cardiovasculares, estresse e erros ocupacionais^(7,8)^, ainda existem poucas investigações que ampliem essa análise para outros grupos laborais expostos a turnos similares.

Estudos epidemiológicos amplamente reconhecidos indicam que a duração ideal do sono para adultos saudáveis deve variar entre sete e nove horas por noite, com uma faixa considerada adequada entre seis e dez horas, a depender das necessidades individuais, idade, estilo de vida e condições de saúde^(9,10)^. A privação de sono crônica, ou a interrupção constante do ciclo de sono, pode ter implicações significativas para a saúde física e mental, contribuindo para o aumento do risco de doenças cardiovasculares, metabólicas, distúrbios cognitivos e diminuição da qualidade de vida^(11)^.

Contudo, quando se observa a realidade dos profissionais de saúde, em especial os enfermeiros, essa recomendação não é comumente alcançada. Estudo anterior evidenciou que a média de horas de sono dos enfermeiros é de apenas seis horas por noite, quantidade inferior ao mínimo recomendado para o bem-estar e o desempenho adequado^(12)^. Isso ocorre devido à natureza das longas e irregulares jornadas de trabalho, especialmente aquelas que envolvem turnos noturnos, que dificultam a manutenção de padrões adequados de sono e, por conseguinte, impactam negativamente a saúde e o rendimento profissional^(4,5)^.

## OBJETIVOS

Estimar a prevalência de privação de sono entre técnicos de enfermagem atuantes em turnos hospitalares e identificar os fatores associados a essa condição.

## MÉTODOS

### Aspectos éticos

Este estudo seguiu rigorosamente os princípios éticos estabelecidos para pesquisas com seres humanos. Todos os participantes foram convidados a participar de forma voluntária, os quais foram devidamente informados sobre os objetivos, procedimentos, potenciais riscos e benefícios do estudo. Antes da coleta de dados, cada profissional assinou o Termo de Consentimento Livre e Esclarecido, o que garantiu sua concordância e compreensão sobre a pesquisa, conforme preconiza a Resolução n^o^ 466/12 do Conselho Nacional de Saúde.

Além disso, o estudo foi submetido e aprovado pelo Comitê de Ética em Pesquisa da Universidade de Rio Verde, sob o Certificado de Apresentação para Apreciação Ética, com o cumprimento das normativas éticas e científicas vigentes. O sigilo e a confidencialidade das informações dos participantes foram preservados em todas as etapas da pesquisa.

### Desenho, período e local do estudo

O presente estudo, de caráter transversal e analítico, foi conduzido de acordo com as diretrizes da metodologia *STrengthening the Reporting of OBservational studies in Epidemiology*. Investigou a prevalência de privação do sono entre técnicos de enfermagem que atuam em turnos hospitalares e sua relação com condições socioeconômicas, profissionais, comportamentais, nutricionais e fatores relacionados ao sono. A coleta de dados foi realizada por meio de questionários individuais estruturados, com a finalidade de obter informações detalhadas sobre os participantes.

A pesquisa foi conduzida em hospital municipal universitário de um município goiano, em uma unidade hospitalar de referência, no período de março a maio de 2022. O hospital oferece atendimento de média e alta complexidade, e conta com uma equipe multiprofissional qualificada. A estrutura da unidade inclui leitos para internação clínica e cirúrgica, além de serviços de urgência e emergência, tornando-se um ambiente estratégico para o estudo de profissionais submetidos a diferentes turnos de trabalho.

### População ou amostra; critérios de inclusão e exclusão

A população do estudo foi composta por técnicos de enfermagem atuantes em ambiente hospitalar, os quais compuseram um total de 218 profissionais. A amostra foi não probabilística por conveniência, incluindo todos os técnicos de enfermagem presentes no hospital durante o período de coleta de dados. Foram incluídos profissionais de ambos os sexos, com idade entre 18 e 60 anos, enquanto aqueles com menos de seis meses de atividade ininterrupta foram excluídos. No total, participaram 198 voluntários, distribuídos entre os três turnos de trabalho.

### Protocolo do estudo

Os participantes foram abordados durante seus períodos de descanso em uma sala disponibilizada pela administração do hospital, com privacidade e conforto para a aplicação do questionário de pesquisa.

O questionário utilizado foi padronizado, pré-testado e autoaplicável, abrangendo variáveis demográficas, socioeconômicas, nutricionais, comportamentais, profissionais e relacionadas à saúde mental e ao sono.

A variável de desfecho foi a privação do sono, mensurada a partir do componente de duração do sono do *Pittsburgh Sleep Quality Index* (PSQI). Esse componente foi determinado por meio de questões que investigam o horário habitual de dormir, o horário de despertar e o tempo necessário para adormecer, permitindo o cálculo da média de horas dormidas por noite^(13)^. A privação do sono foi categorizada conforme critérios amplamente adotados na literatura: indivíduos com tempo médio de sono inferior a cinco horas por noite (< cinco horas) foram classificados como privados de sono, enquanto aqueles com tempo igual ou superior a cinco horas (≥ cinco horas) foram considerados com sono adequado^(14)^.

As variáveis exploratórias foram agrupadas em categorias temáticas para fins de organização e análise: (i) demográficas, socioeconômicas e profissionais; (ii) nutricionais; (iii) comportamentais; (iv) saúde mental; e (v) relacionadas ao sono. (i) Demográficas, socioeconômicas e profissionais: sexo (masculino; feminino), estado civil (com companheiro; sem companheiro), escolaridade (< 15 anos de estudo; ≥ 15 anos), tempo médio de profissão (em anos) e carga horária diária de trabalho (≤ 12 horas; > 12 horas). (ii) Nutricionais: consumo de frutas, legumes e verduras (> cinco porções/dia - adequado; < cinco porções/dia - inadequado)^(15)^, consumo de *fast food* (sim; não), peso e altura autorreferidos e Índice de Massa Corporal (IMC). (iii) Comportamentais: consumo de álcool (sim: já consumiu; não: nunca consumiu ou deixou de beber), consumo de café (sim; não), tabagismo (fumantes atuais; não fumantes/ex-fumantes), nível de dependência de internet (leve; moderada/alta), avaliada pelo *Internet Addiction Test* ^(16)^, e risco de dependência de álcool (baixo risco; risco de dependência), categorizado pela escala *Alcohol Use Disorders Identification Test*. (iv) Saúde mental: sintomas de depressão, ansiedade e estresse avaliados pela *Depression, Anxiety and Stress Scale-21* ^(17)^ e categorizados como baixo ou alto risco, além da autopercepção de saúde (boa/muito boa; ruim/péssima). (v) Relacionadas ao sono: qualidade do sono avaliada pelo PSQI (boa; má)^(13)^ e sonolência diurna excessiva (SDE), mensurada pela *Epworth Sleepiness Scale* e categorizada como presença (> 10) ou ausência (< 10)^(15,18)^.

### Análise dos resultados e estatística

A entrada de dados foi realizada no *software* EpiData 3.1, com dupla digitação para minimizar erros. A validação e a análise de dados ocorreram no *software* Stata 15.0. Inicialmente, as informações foram organizadas no *Microsoft Excel* ^®^ 2013, e o tratamento estatístico foi conduzido no *Statistical Package for the Social Sciences* ^®^.

Foram realizadas análises estatísticas descritivas para caracterizar a amostra e as variáveis do estudo. Utilizaram-se frequências absolutas e relativas. Para identificar os fatores associados à privação do sono, aplicaram-se regressões logísticas bivariada e multivariada. Inicialmente, cada variável independente foi analisada isoladamente na regressão logística bivariada para verificar sua relação com a privação do sono. As variáveis que apresentaram um valor de p inferior a 0,2 foram selecionadas para compor o modelo multivariado.

Posteriormente, a regressão logística multivariada foi conduzida pelo método *forward*. Incluíram-se progressivamente as variáveis independentes selecionadas na etapa anterior (p < 0,2). Essa abordagem possibilitou a avaliação do efeito combinado das variáveis sobre a privação do sono, considerando os potenciais fatores de confusão. As estimativas de associação foram expressas em Razões de Chances (*Odds Ratios* - OR) com seus respectivos Intervalos de Confiança de 95% (IC95%). Para determinar a significância estatística das associações no modelo multivariado, foi adotado o critério de p < 0,05.

## RESULTADOS

A amostra foi composta por 198 participantes, em sua grande maioria mulheres (95,5%), com média de idade de 37,2 anos. A Tabela 1 apresenta os dados da privação de sono e sua associação com as características sociodemográficas, profissionais, nutricionais, comportamentais, relacionadas à saúde mental e ao sono dos técnicos de enfermagem. A maioria dos indivíduos se declarou estar em união estável (60,6%). Ademais, 68,2% possuíam até 15 anos de estudo. A média de tempo de profissão foi de 7,5 anos. Em relação ao comportamento alimentar, 59,1% dos participantes apresentaram consumo adequado de frutas, verduras e legumes, enquanto 68,2% consumiam *fast food*. A prevalência de consumo de álcool foi alta, com 69,7% dos participantes relatando o hábito, e 83,3% consumiam café regularmente. A maioria dos participantes (93,9%) era tabagista ou ex-tabagista, e 62,1% apresentavam sobrepeso ou obesidade (IMC ≥ 25). Além disso, 42,4% dos participantes relataram sintomas de ansiedade, e 12,1% apresentaram sintomas depressivos. A qualidade do sono se apresentou predominantemente ruim, com 93,9% dos participantes reportando essa condição. A prevalência de privação de sono entre os participantes foi de 43,9%, o que destaca a magnitude desse problema na rotina desses profissionais.

**Tabela 1:** Frequência da privação do sono e associação com as características sociodemográficas, profissionais, nutricionais, comportamentais, relacionadas à saúde mental e ao sono de técnicos de enfermagem, Goiás, Brasil, 2022

Variáveis	Total	Privação de sono	
	n (%)	OR (IC95%)	Valor de *p*
Sexo			
Feminino	189 (95,5%)	1	0,174
Masculino	9 (4,5%)	2,67 (0,65-10,98)	
Estado civil			
Com companheiro	78 (39,4%)	1	0,211
Sem companheiro	120 (60,6%)	1,45 (0,81-2,59)	
Escolaridade			
Menos de 15 anos	135 (68,2%)	1	0,476
15 anos ou mais	63 (31,8%)	1,24 (0,68-2,27)	
Tempo de profissão	7,5±7,0	0,98 (0,95-1,03)	0,500
Horas/dia trabalho			
≤ 12 horas	132 (66,7%)	1	0,035^*^
> 12 horas	66 (33,3%)	1,90 (1,05-3,47)	
Consumo de frutas, verduras e legumes			
Adequado (> cinco porções/dia)	117 (59,1%)	1	0,063
Inadequado (< cinco porções/dia)	81 (40,9%)	1,72 (0,97-3,06)	
Consumo de *fast food*			
Não consome	63 (31,8%)	1	0,834
Consome	135 (68,2%)	1,07 (0,58-1,95)	
Consumo de álcool			
Não	60 (30,3%)	1	0,462
Sim	138 (69,7%)	1,26 (0,68-2,33)	
Consumo de café			
Não	33 (16,7%)	1	0,038^*^
Sim	165 (83,3%)	2,39 (1,05-5,45)	
Tabagista			
Não/ex-tabagista	12 (6,1%)	1	0,524
Sim	186 (93,9%)	1,15 (0,75-1,76)	
IMC			
< 24,9	75 (37,9%)	1	0,008^*^
25 a 29,9 /> 30	123 (62,1%)	1,45 (0,25-1,81)	
Nível de dependência da internet			
Leve	183 (92,4%)	1	0,200
Moderado/alta	15 (7,6%)	2,02 (0,69-5,91)	
Risco de dependência do álcool			
Baixo risco	165 (83,3%)	1	0,038^*^
Risco de dependência	33 (16,7%)	0,42 (0,18-0,95)	
Depressão			
Não	174 (87,9%)	1	0,194
Sim	24 (12,1%)	1,72 (0,76-3,89)	
Ansiedade			
Não	114 (57,5%)	1	0,001^*^
Sim	84 (42,4%)	2,62 (1,47-4,66)	
Auto percepção saúde			
Boa/muito boa	141 (71,2%)	1	0,741
Ruim/péssima	57 (28,8%)	0,90 (0,48-1,68)	
Qualidade do sono (PSQI)			
Boa qualidade	12 (6,1%)	1	0,001^*^
Má qualidade	186 (93,9%)	3,29 (0,25-6,53)	
SDE			
Sem SDE	117 (59,7%)	1	0,905
Com SDE	81 (40,9%)	1,03 (0,58-1,83)	

*IMC - Índice de Massa Corporal; SDE - sonolência diurna excessiva; PSQI - Pittsburgh Sleep Quality Index; OR - Odds Ratio; IC95% - Intervalo de Confiança de 95%;*

* Associação estatisticamente significativa.

A análise multivariada realizada identificou uma série de fatores associados à privação de sono, que, por sua vez, revelaram relações significativas com diversas variáveis sociodemográficas, comportamentais e de saúde. Entre os principais determinantes identificados, destacam-se a jornada de trabalho diária, o padrão alimentar relacionado ao consumo de frutas, legumes e verduras, o consumo de café, o IMC e a presença de sintomas ansiosos. Embora o sexo e o risco de dependência de álcool também tenham sido analisados, esses fatores não apresentaram associação estatisticamente significativa com a privação de sono. Portanto, sugere-se que a influência desses aspectos pode ser secundária em relação a outros determinantes (Tabela 2).

**Tabela 2:** Associação das variáveis com privação do sono entre técnicos de enfermagem na análise de regressão logística multivariada, Goiás, Brasil, 2022

Variáveis	Privação de sono	
	OR (IC95%)	Valor de *p*
Sexo		
Feminino	1	0,137
Masculino	3,41(0,68-17,17)	
Horas/dia trabalho		
≤ 12 horas	1	0,001^*^
> 12 horas	3,60(1,72-7,56)	
Consumo de frutas, verduras e legumes		
Adequado (> cinco porções/dia)	1	0,024^*^
Inadequado (< cinco porções/dia)	2,25(1,11-4,54)	
Consumo de café		
Não	1	0,031^*^
Sim	3,14(1,11-8,89)	
IMC		
< 24,9	1	0,001^*^
25 a 29,9/30	1,27(0,13-0,56)	
Risco de dependência de álcool		
Baixo risco	1	0,059
Risco de dependência	0,41(0,16-1,03)	
Ansiedade		
Não	1	0,001^*^
Sim	6,58(3,07-14,11)	
Qualidade do sono (PSQI)		
Boa qualidade	1	0,001^*^
Má qualidade	3,36 (1,23-5,57)	

*IMC - Índice de Massa Corporal; PSQI - Pittsburgh Sleep Quality Index; OR - Odds Ratio; IC95% - Intervalo de Confiança de 95%;*

* Associação estatisticamente significativa.

A jornada de trabalho diária foi um dos fatores mais fortemente associados à privação de sono. Indivíduos que trabalhavam mais de 12 horas por dia apresentaram um risco 3,6 vezes maior de sofrer de privação de sono em comparação aos que trabalhavam até 12 horas (OR = 3,60; IC95%: 1,72-7,56; p = 0,001). Esse dado sugere que jornadas de trabalho prolongadas têm um impacto significativo na qualidade do sono.

O consumo inadequado de frutas, legumes e verduras também esteve relacionado à privação de sono. Os participantes com uma dieta inadequada nesse aspecto apresentaram uma chance 2,25 vezes maior de experimentar privação de sono em relação aos que consumiam uma quantidade adequada de frutas, legumes e verduras (OR = 2,25; IC95%: 1,11-4,54; p = 0,024), indicando que a alimentação desempenha um papel importante no bem-estar do sono.

O consumo de café foi outro fator significativo, sendo que aqueles que consumiram café apresentaram um risco 3,14 vezes maior de sofrer de privação de sono em comparação aos que não consumiram a bebida (OR = 3,14; IC95%: 1,11-8,89; p = 0,031). Isso sugere que o consumo de café pode afetar negativamente o sono, possivelmente devido ao efeito estimulante da cafeína.

Em relação ao IMC, os participantes com valores mais altos apresentaram maior risco de privação de sono. Indivíduos com IMC superior a 24,9 apresentaram uma chance significativamente maior de ter problemas com o sono, com uma OR de 1,27 (IC95%: 0,13-0,56; p = 0,001) em comparação aos que tinham IMC abaixo de 24,9.

A presença de ansiedade foi um dos fatores mais fortemente associados à privação do sono, com indivíduos ansiosos apresentando uma OR significativamente maior (OR = 6,58; IC95%: 3,07-14,11; p = 0,001). Além disso, indivíduos com má qualidade do sono apresentaram maior prevalência de privação do sono (OR = 3,36; IC95%: 1,23-5,57; p = 0,001).

## DISCUSSÃO

A alta prevalência de privação do sono observada entre os técnicos de enfermagem que trabalham em turnos deste hospital universitário municipal em Goiás (43,9%) está em conformidade com trabalhos anteriores que documentam essa condição entre profissionais de saúde, especialmente em ambientes de cuidados críticos. Autores relataram taxas de prevalência de privação do sono que variam de 20% a 51%, semelhantes aos 43,9% encontrados neste estudo^(19,20)^. Esses achados reforçam a ideia de que fatores ocupacionais, como turnos noturnos, jornadas de trabalho prolongadas e altas demandas emocionais e cognitivas, enfrentados pelos profissionais de saúde, são determinantes significativos da privação do sono. A literatura sugere que, devido à sobrecarga de trabalho e às exigências emocionais, os profissionais de saúde frequentemente apresentam fragmentação do sono e redução da qualidade do descanso^(21,22)^.

A privação do sono entre profissionais de saúde tem sido amplamente associada a diversos desfechos adversos, como fadiga, redução da capacidade cognitiva e aumento do risco de erros médicos^(23)^. Estudo identificou que os profissionais de enfermagem estão entre os mais afetados pela privação do sono em comparação com outras categorias ocupacionais, devido à natureza do trabalho por turnos e à necessidade constante de adaptação a horários irregulares. Esses achados destacam a importância de considerar a estrutura do trabalho, incluindo a flexibilidade dos turnos e o gerenciamento do estresse, para reduzir a prevalência da privação do sono e seus efeitos sobre a saúde dos trabalhadores e a segurança dos pacientes^(24)^.

O estudo revelou que trabalhar mais de 12 horas por dia esteve significativamente associado à privação do sono. Esse achado é consistente com outras pesquisas que indicam que os turnos prolongados e a necessidade do trabalho noturno impactam negativamente os padrões de sono^(9,10)^. Em particular, enfatiza-se que o estresse crônico associado aos serviços hospitalares, combinado com longas jornadas, contribui para a fragmentação do sono e o aumento da privação do sono, o que leva a um ciclo vicioso de fadiga e exaustão^(6)^.

Outro fator relevante identificado neste estudo foi a associação entre o consumo inadequado de frutas, verduras e legumes e a privação do sono, embora com uma tendência à significância estatística (p = 0,063). Dietas ricas em micronutrientes como magnésio, triptofano e vitaminas do complexo B desempenham um papel fundamental na regulação do ciclo circadiano e na produção de neurotransmissores relacionados ao sono, como serotonina e melatonina^(25)^. Dietas pobres nesses nutrientes podem prejudicar a qualidade do sono. Assim, intervenções nutricionais voltadas ao aumento do consumo de frutas, verduras e legumes podem ser eficazes na melhoria do sono nessa população^(26)^.

O consumo de cafeína também foi identificado como um fator significativamente associado à privação do sono. A cafeína é conhecida por seu efeito estimulante, que pode prolongar o estado de vigília e reduzir a eficiência do sono, especialmente quando consumida em horários inadequados^(27)^. Esses achados estão alinhados com outro estudo que documenta a relação entre o consumo de cafeína e distúrbios do sono^(28)^. Tais achados reforçam a necessidade de orientação sobre o impacto do consumo de estimulantes no bem-estar dos profissionais de saúde.

O achado de que indivíduos com IMC acima de 24,9 kg/m^2^ apresentaram maior risco de privação do sono é consistente com a literatura, que sugere uma relação bidirecional entre excesso de peso e distúrbios do sono. A obesidade pode afetar negativamente a qualidade do sono por meio de mecanismos fisiológicos, como inflamação sistêmica de baixo grau e alterações hormonais, que impactam a regulação do ciclo circadiano e a homeostase do sono^(29)^. Além disso, indivíduos com sobrepeso e obesidade frequentemente apresentam maior risco de distúrbios respiratórios do sono, como a síndrome da apneia obstrutiva do sono, que leva à fragmentação do sono e à SDE^(30)^.

Por outro lado, evidências sugerem que a privação do sono pode contribuir para o ganho de peso, pois está associada a alterações no metabolismo energético e no apetite, o que culmina em maior ingestão calórica, redução da saciedade e desequilíbrio na secreção de hormônios reguladores, como leptina e grelina^(31)^. Assim, o efeito observado neste estudo pode fazer parte de um ciclo de retroalimentação, no qual o aumento do IMC contribui para a piora da qualidade do sono, enquanto a privação do sono favorece o ganho de peso.

A relação entre privação do sono e saúde mental foi um dos achados mais evidentes, com uma associação significativa entre a presença de ansiedade e a privação do sono. A literatura indica que a relação entre transtornos emocionais e sono é bidirecional: a ansiedade pode dificultar a manutenção do sono e, por sua vez, a privação do sono pode exacerbar os sintomas de ansiedade^(11)^. Técnicos de enfermagem que atuam em hospitais de média e alta complexidade estão expostos a altas demandas emocionais e estresse constante, o que pode explicar a alta prevalência de transtornos emocionais nessa população. Portanto, cuidar da saúde mental desses profissionais é crucial para melhorar a qualidade do sono e, consequentemente, a qualidade do atendimento prestado.

O risco de dependência alcoólica foi inversamente associado à privação do sono, com menor prevalência de privação do sono entre aqueles com maior risco de dependência (p = 0,038). Embora o álcool tenha um efeito sedativo, há evidência de que ele prejudica a qualidade do sono ao reduzir o sono REM e aumentar a fragmentação do sono noturno, o que, a longo prazo, pode agravar os distúrbios do sono^(32)^.

A associação entre má qualidade do sono e privação do sono observada neste estudo (OR = 3,36; IC95%: 1,23-5,57; p = 0,001) reforça achados anteriores que indicam que indivíduos com pior qualidade do sono têm maior probabilidade de desenvolver privação do sono. A literatura sugere que a má qualidade do sono está frequentemente relacionada a fatores como fragmentação do sono, dificuldade para iniciar ou manter o sono e despertares frequentes, resultando em menor eficiência do sono e, consequentemente, em um tempo total de descanso insuficiente para a recuperação fisiológica e mental adequada^(33)^.

Estudos anteriores sugerem que a má qualidade do sono pode estar associada a múltiplos fatores, o que inclui estresse, condições ambientais desfavoráveis, hábitos inadequados de sono e demandas ocupacionais excessivas^(34,35)^. Indivíduos com padrões de sono irregulares, como trabalhadores noturnos ou aqueles submetidos a longas jornadas, frequentemente relatam pior qualidade do sono e maior privação do sono, o que pode contribuir para alterações metabólicas, déficits cognitivos e piora da saúde mental^(23)^.

A relação entre qualidade do sono e privação do sono é bidirecional. A má qualidade do sono pode contribuir para a privação do sono ao reduzir a eficiência do descanso e comprometer a restauração fisiológica e cognitiva^(36)^. Por sua vez, a privação crônica do sono pode exacerbar a fragmentação do sono e reduzir sua profundidade, aumentando o risco de distúrbios como insônia e SDE^(37)^.

Em resumo, os achados deste estudo destacam a importância de abordar a privação do sono entre técnicos de enfermagem sob uma perspectiva multidisciplinar, ao considerar fatores ocupacionais, dietéticos, de saúde mental e comportamentais. A implementação de políticas de saúde ocupacional voltadas à melhoria da qualidade do sono, como a reestruturação dos horários de trabalho, a promoção de uma alimentação equilibrada e o fornecimento de suporte psicológico, pode contribuir significativamente para a redução da privação do sono e de seus impactos na saúde e no desempenho profissional.

### Limitações do estudo

Este estudo apresenta algumas limitações que devem ser consideradas ao interpretar os resultados. Primeiramente, sua natureza transversal impede a determinação de relações causais entre privação do sono e fatores associados, o que limita os achados a associações estatísticas. Além disso, a coleta de dados foi realizada por meio de questionários autorrelatados, sujeitos a viés de memória e resposta, especialmente em variáveis subjetivas como qualidade do sono e sintomas de ansiedade. Outra limitação diz respeito à amostra, pois o estudo foi conduzido em um único município do sudoeste goiano, o que pode restringir a generalização dos achados para outras populações de técnicos de enfermagem com diferentes condições de trabalho e contextos socioeconômicos.

Para superar as limitações deste estudo, pesquisas futuras poderiam adotar um delineamento longitudinal, possibilitando o estabelecimento de relações causais entre as variáveis e a privação do sono.

Apesar dessas limitações, o estudo apresenta pontos fortes relevantes, pois o uso de instrumentos validados para avaliar o sono e a saúde mental fortalece a robustez dos dados, permitindo comparações com estudos anteriores. Além disso, ao abordar um grupo profissional altamente suscetível à privação do sono devido às características laborais, o estudo contribui para a compreensão dos fatores que impactam a saúde desses trabalhadores e fornece subsídios para o desenvolvimento de estratégias de intervenção. Outro aspecto positivo é a análise detalhada de múltiplos fatores associados à privação do sono, que oferece uma visão abrangente das influências ocupacionais, comportamentais e emocionais sobre esse desfecho.

### Contribuições para a área da enfermagem, saúde ou políticas públicas

Os achados deste estudo contribuem para a prática da enfermagem ao evidenciarem a elevada prevalência de privação de sono entre os técnicos de enfermagem em turnos hospitalares e ao destacarem os fatores ocupacionais, nutricionais, comportamentais e emocionais associados a essa condição. Ao delimitar variáveis diretamente relacionadas à jornada de trabalho, ao padrão alimentar, ao consumo de substâncias estimulantes e à saúde mental, o estudo oferece subsídios práticos para o planejamento de estratégias de promoção da saúde do trabalhador.

Na prática assistencial, os achados reforçam a necessidade de programas educativos e preventivos voltados ao manejo da privação de sono, contemplando desde intervenções organizacionais sobre a carga horária de trabalho até ações de incentivo à alimentação equilibrada, moderação no consumo de estimulantes e suporte psicossocial. Tais medidas podem contribuir não apenas para a melhoria da qualidade do sono, mas também para a segurança do cuidado prestado e para a redução do desgaste físico e emocional dos profissionais.

Em nível institucional e de políticas públicas, os dados podem orientar gestores na formulação de diretrizes que reconheçam a privação de sono como um importante problema de saúde ocupacional. A incorporação desse tema em agendas de saúde coletiva favorece a criação de ambientes laborais mais sustentáveis, com repercussões positivas para a qualidade de vida dos profissionais e, indiretamente, para a qualidade da assistência em saúde. Além disso, os resultados abrem espaço para novos caminhos de pesquisa que aprofundem a compreensão dos mecanismos de interação entre fatores ocupacionais, nutricionais e psicológicos na privação de sono em profissionais de saúde.

## CONCLUSÕES

Este estudo evidenciou uma alta prevalência de privação de sono (43,9%) entre os participantes, o que é consistente com a literatura existente, que frequentemente aponta jornadas de trabalho prolongadas, hábitos alimentares inadequados, questões relacionadas à saúde mental e qualidade do sono como determinantes significativos para a privação do sono.

A análise demonstrou que a jornada de trabalho diária, o consumo de frutas, legumes e verduras, o consumo de café, o IMC e a presença de sintomas ansiosos são fatores significativamente relacionados à privação de sono. Entre esses, a jornada de trabalho prolongada foi o fator mais fortemente associado, com indivíduos que trabalhavam mais de 12 horas por dia apresentando um risco significativamente maior de sofrer de privação de sono. Além disso, uma dieta inadequada e o consumo de café também se mostraram fatores de risco, reforçando a importância de hábitos alimentares saudáveis e da moderação no consumo de substâncias estimulantes.

A presença de ansiedade foi outro fator de destaque, com indivíduos ansiosos apresentando uma probabilidade substancialmente maior de sofrer de privação de sono. Esse achado destaca a importância de abordagens psicológicas, como o gerenciamento do estresse e a promoção do bem-estar emocional, no manejo da privação de sono. Embora o sexo e o risco de dependência de álcool não tenham mostrado uma associação significativa, os resultados sugerem que esses fatores podem ter um impacto secundário, sendo superados por outros determinantes mais diretamente relacionados à saúde do sono.

Esses achados indicam que a prevenção e o tratamento da privação de sono devem envolver estratégias multifacetadas que considerem não apenas as condições de trabalho, mas também os hábitos alimentares, o consumo de substâncias e fatores psicológicos. Intervenções que abordem essas variáveis podem contribuir para a melhoria da qualidade do sono e, consequentemente, para a saúde e o bem-estar dos profissionais de enfermagem, especialmente aqueles que trabalham em turnos em ambientes críticos.

## Data Availability

Os dados de pesquisa só estão disponíveis mediante solicitação.
